# Systematic review on biomechanical effects of high-velocity, low amplitude spinal manipulation

**DOI:** 10.1371/journal.pone.0328048

**Published:** 2025-07-18

**Authors:** Anke Langenfeld, Mirjam Baechler, Jaap Swanenburg, Malin Mühlemann, Luana Nyirö, Daniel Streuli, Brigitte Wirth, Petra Schweinhardt

**Affiliations:** 1 Integrative Spinal Research Group, Department of Chiropractic Medicine, University Hospital Balgrist and University of Zurich, Zurich, Switzerland; 2 Department of Chiropractic Medicine, University Hospital Balgrist and University of Zurich, Zurich, Switzerland; 3 Faculty of Medicine, Institute of Aerospace Medicine, University of Zurich, Zurich, Switzerland; National Institute of Health and Medical Research: INSERM, FRANCE

## Abstract

**Background:**

Spinal manipulative therapy uses high-velocity low-amplitude (HVLA) thrusts which are clinically effective, but underlying mechanisms are still unknown.

**Objective:**

To summarize the evidence for biomechanical effects of HVLA thrusts in asymptomatic and symptomatic humans as well as for a possible link between biomechanical effects and clinical effectiveness.

**Study design:**

Systematic review of randomized controlled trials.

**Methods:**

An information specialist conducted systematic literature searches in six databases [Medline (OvidSP), Premedline (PubMed), CINAHL, EMBASE, Cochrane, and Biosis]. Studies were selected by two authors and classified using the revised Cochrane risk-of-bias tool for randomized trials (RoB 2). Results were qualitatively summarized per biomechanical output [range of motion (ROM) according to the spinal region where HVLA thrusts were applied, facet joint gapping, and spinal stiffness].

**Results:**

The thirty-three included studies were heterogeneous regarding participant characteristics, intervention frequency and outcomes. Twenty-seven studies reported on the effects of HVLA thrusts on spinal ROM. There is evidence increased cervical ROM following cervical HVLA thrusts on cervical ROM [9/15 studies with a positive treatment effect (5 low risk of bias/4 some concern) and increased cervical ROM following thoracic HVLA thrusts [8/8 studies (4 low risk of bias/4 some concern)]. The effects of a thoracic and lumbar HVLA thrusts on the respective ROM were less clear. Three (2 low risk/1 some concern) studies on facet joint gapping showed increased gapping following HVLA thrusts compared to side-posture positioning, and a single study (some concern) on spinal stiffness showed no effect of HVLA thrusts. Only one study (some concern) linked the biomechanical to clinical outcomes.

**Conclusion:**

HVLA thrusts, either applied to the cervical or the thoracic spine, appear to increase cervical ROM, based on studies with low risk and studies with some concern regarding risk of bias. For all other outcomes, the included studies were too heterogeneous and too few to draw any sound conclusion. Future studies on the biomechanical effects of HVLA thrusts should link the biomechanical to clinical outcomes such as pain and disability.

**PROSPERO registration:**

CRD42018096963.

## Introduction

High-velocity, low-amplitude (HVLA) thrusts are widely used by chiropractors, physical therapists, osteopaths, and medical doctors [1]. SMT using HVLA thrusts are thought to be clinically effective and is recommended in recent guidelines for the management of acute and chronic low back pain (LBP) [2], neck pain [3], cervical [4], and lumbar radiculopathy [5]. The effects of HVLA thrusts on pain and function in patients with neck and LBP are comparable to other recommended treatment options, like medication and exercise [6–8].

HVLA thrusts can be applied manually, by a hand-held instrument (instrument-applied manipulation, IAM), or by a robot-like apparatus [9,10]. The manual procedure is characterized by a force application through the hands of the practitioner directed to the intervertebral joints [11–13]. The target joint is brought to the end of its range of motion (ROM) by the application of a preload force, followed by a high-velocity thrust over a short-amplitude [9,14]. This application targets the intervertebral joint but the forces are also transmitted to the surrounding soft tissues [15]. For IAM, which is clinically effective and safe, a force is delivered to the spine by a spring-loaded hand-held instrument with a small rubber tip [16]. For research, a robot-like apparatus has been developed that can apply a thrust to the human spine in a standardized manner with different force-time profile settings [10].

The effects of HVLA thrusts have been categorized as neurophysiological or biomechanical, e.g., increased range of motion, or reduced muscle activity, with the overall effects being most likely a complex interplay between the two [15,17,18].

Previous systematic reviews have investigated the biomechanical effects of HVLA thrusts directed to the cervical or thoracic spine on cervical range of motion (ROM) [19–21]. HVLA thrusts directed to the cervical or thoracic spine were shown to increase cervical ROM [19–21]. In contrast, systematic reviews on lumbar HVLA thrusts found no effect on lumbar ROM [19]. No review has examined the effects of thoracic HVLA thrusts on thoracic ROM or other biomechanical outcomes. Additionally, information linking biomechanical effects of HVLA thrusts to clinical outcome is missing [1,9].

Therefore, this systematic review aimed to summarize the evidence for biomechanical effects of an HVLA thrusts in asymptomatic and symptomatic humans as well as to explore a possible link between these biomechanical effects and clinical effectiveness.

## Materials and methods

This systematic review is reported in accordance with the Preferred Reporting Items of Systematic Review and Meta-Analysis guidelines [22]. The protocol of this systematic review was registered at the International Prospective Register of Systematic Reviews (PROSPERO: CRD42018096963).

### Search strategy

An a priori search strategy was agreed on at the beginning of the project by three of the authors (DS, PS, BW). The initial literature search was undertaken by an information specialist at the University of Zurich, Zurich, Switzerland. All articles published until June 14, 2018, were included. No time restriction regarding the start date was applied. The literature search was updated three times for the times June 2018 – October 2020, October 2020 – March 2023 and March 2023 – November 2024 by the same information specialist using the same search strategy (see appendix). Databases were Medline, CINAHL, EMBASE, PubMed, Cochrane, and Biosis.

### Inclusion and exclusion criteria

This review focused on peer-reviewed publications reporting on randomized controlled trials in English that investigated biomechanical effects, changes in ROM, changes in the distance between articular surfaces of the zygapophyseal joint [gapping] [23], and resistance to elastic deformation of a joint (stiffness) [24], following manual, instrumented, or apparatus HVLA thrust in symptomatic or asymptomatic humans.

Studies exclusively reporting neurophysiological or subjective outcomes or using any modeling, such as finite element models, were excluded. The review included studies that used HVLA thrusts either as a standalone or combined intervention, provided that the effect of the HVLA thrusts could be isolated [e.g., HVLA thrusts plus electrotherapy versus electrotherapy alone]. Studies involving mobilization were included when mobilization was used as a control intervention against HVLA thrusts. Since the key difference between mobilization and HVLA thrusts is the application of a thrust, mobilization is considered an appropriate control intervention.

### Study selection

After removing duplicate articles, two reviewers independently screened titles and abstracts to determine eligibility. If eligibility could not be determined, the full text was consulted. If articles were deemed eligible during abstract and title screening, the full text was assessed, and data was extracted if included in the review. Discrepancies were resolved through discussion between the two reviewers. If discrepancies could not be resolved, a third reviewer (PS) was consulted.

### Risk of bias assessment

The revised Cochrane risk-of-bias tool for randomized trials (RoB 2, beta version, 15 March 2019) was used to assess the quality of the included studies in five bias categories, i.e., randomization process, deviations from intended interventions, missing outcome data, outcome measurement, and selection of reported results, as well as an overall bias classification. Each of the categories allows for a classification of ‘low risk’, ‘some concerns’, or ‘high risk’ [25]. Two reviewers from the pool [all listed authors] were randomly allocated to studies and independently classified each study, and discrepancies in any of the bias categories were resolved through discussion between the two reviewers. If a consensus could not be reached, a third reviewer (PS) was involved. To determine the best evidence, studies deemed ‘high risk’ were excluded from the narrative synthesis.

### Data extraction

Data to be extracted was defined prior to the study and included subject characteristics, treatment region, description of intervention and control interventions, timing of assessment relative to intervention, biomechanical outcomes, and main biomechanical results. Extracted data was verified by a second researcher and summarized per biomechanical output (range of motion (ROM) according to the spinal region where HVLA thrust was applied, facet joint gapping, and spinal stiffness).

## Results

### Study selection and study quality

**Fig 1:**
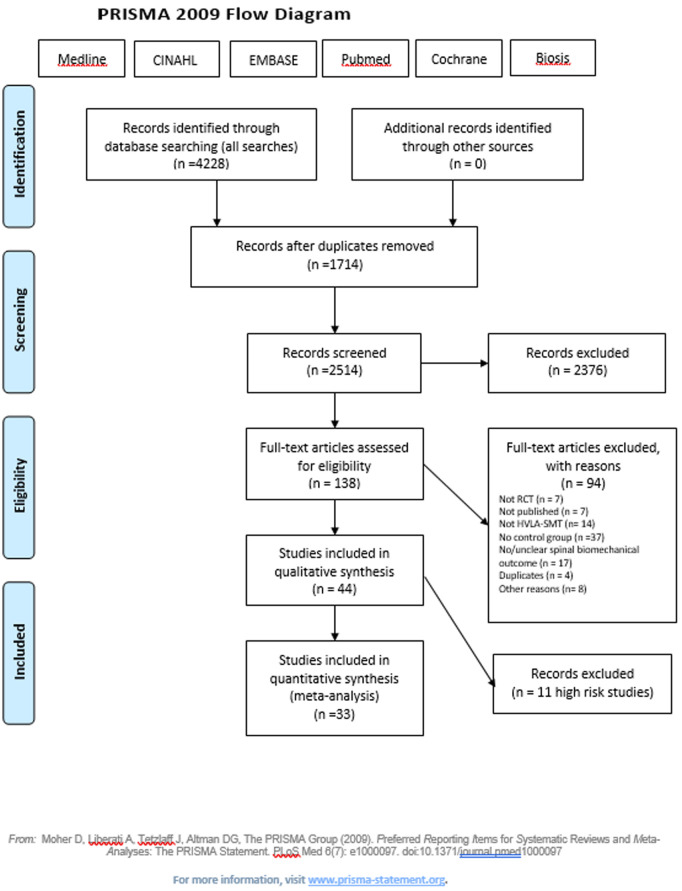
Prisma flow diagram for the whole study including searches 2018, 2020, 2023 and 2024.

Titles and abstracts were screened based on predefined inclusion and exclusion criteria by two independent reviewers. The PRISMA flowchart outlines the selection process (Fig 1). A total of 4,228 studies were identified, 1,714 (40.5%) duplicate records were removed, 2,514 (59.4%) records were screened, and 2,376 (56.1%) records were excluded. The remaining 138 (3.2%) full-text articles were assessed, leading to the exclusion of 94 (2.2%) studies. The assessment of study quality resulted in 16 (0.3%) articles with ‘low risk’ [26–41], 17 (0.4%) articles with ‘some concerns’ (Fig 1), and 11 articles (0.2%) with ‘high risk’ [42–52]. The level of agreement of ‘high risk’ (excluded studies) vs ‘low risk’ or ‘some concerns’ (included studies) was moderate (ĸ = 0.58). A quantitative analysis (meta-analysis) was not performed due to the high heterogeneity of data with regard to participant characteristics, intervention frequency, and outcomes.

**Fig 2:**
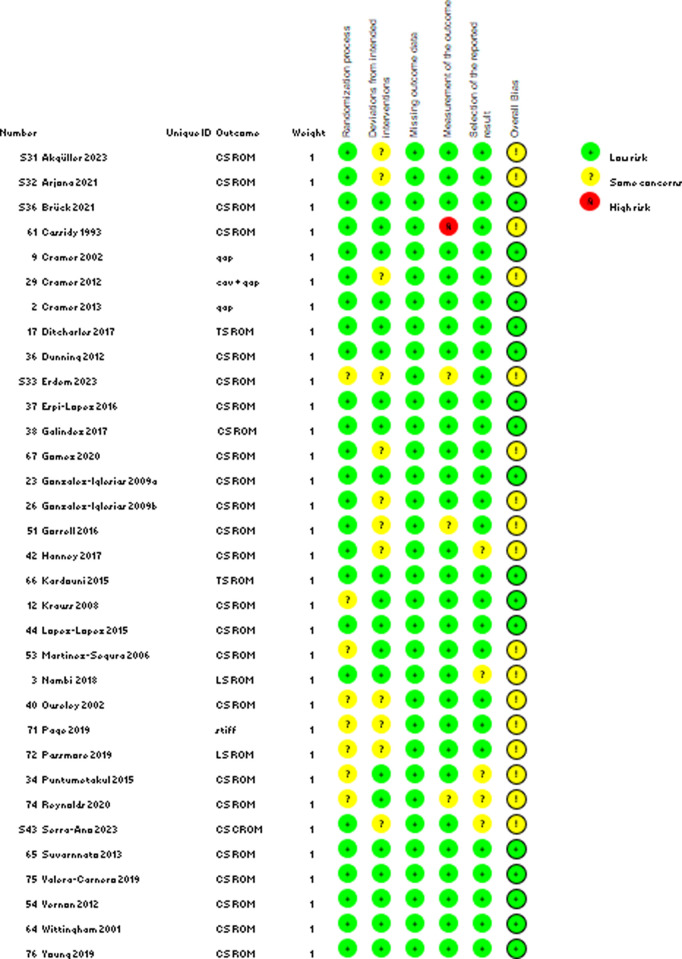
Results of the quality assessment using the RoB 2 tool including studies with low risk and some concerns [high-risk studies excluded].

Thus, after the exclusion of the high-risk publications, 33 publications were included in the qualitative synthesis (Fig 2).

The majority of the included studies (27/33) reported on the effects of HVLA thrusts on spinal ROM. These effects can be categorized as *local* effects where HVLA thrusts are applied in the same spinal region in which ROM is measured or *regional* effects where HVLA thrusts are applied in a region distant to where ROM is measured. Four studies reported on the effects of HVLA thrusts on gapping of the zygapophyseal [facet] joints [26,27,53] and one on spinal stiffness [54]. Post-treatment measurement time points varied. Eighteen (54%) studies performed measurements pre-treatment and immediately post-treatment [28,29,32–35,37–40,55–62]. Other studies measured 24 hours after treatment [61], 48 hours after treatment [31], one week after treatment [41], after the fifth visit [54], or at the end of each treatment phase [36]. Additionally, if treatment was applied over a period of time, measurements were taken at the end of the treatment series [37,63] (Tables 1–3).

**Table 1:** Local effects of HVLA thrust on range of motion cervical spine.

Autor, Year	Participants [A]Symptomatic Age Number	Treatment region/ Region of interest	Intervention	Control	Time to assessment [relative to intervention]	Biomechanical outcome parameters	Main biomechanical results	RoB Rating
**Cervical spine**								
Serra-Ano et al. [2023]	50 participants with acute and chronic non-specific neck pain [minimum duration of the symptoms being 1 month] Group 1 [n = 25: mean age 33.40 ± 12.06] Group 2 [n = 23: mean age 32.00 ± 12.06]	Cervical spine [C1-C7]	Group 1: [Single session] High-velocity and low-amplitude cervical manipulation with two manipulation attempts per vertebral level identified. Max. of three manipulations	Group 2: [Single session] Sham hands-on technique and pressure-less touches with the physiotherapist`s hands on several head and shoulder areas for 10 minutes. Light superficial touches were applied on standardized anatomic areas for two minutes.	Before and 5 minutes after treatment	Active cervical range of motion in each movement direction [flexion, extension, right and left lateral bending, right and left rotation]	A single session of cervical manipulation had no significant impact on any study biomechanical variables, except for right -side bending [1.97°] and left rotation [1.95°]. No improvement in any ROM in group 2. Effect size right-side bending group 1: 0.35 Effect size left rotation group 1: 0.30 Minimal detectable change not overcome.	**Some concerns**
Akgüller et al. [2023]	60 volunteers [both sexes] with mechanical neck pain lasting 3 months 20-50 years [Mean age: 31.45 ± 7.31] Group 1 [n = 20: mean age: 31.00 ± 7.10] Group 2 [n = 20: mean age: 31.65 ± 8.38] Group 3 [n = 20: mean age: 31.70 ± 6.71]	Cervical spine [C2-C7]	Group 1: Cervical thrust manipulation Group 3: Cervical thrust and exercise Twice a week for six weeks	Group 2: Exercise program consisting of progressive stabilization and exercise was performed by group two under supervision. Twice a week for six weeks	Immediately posttreatment [Six weeks after baseline]	Active neck flexion-extension right-left lateral flexion and right-left rotation ROMs were assessed with the CROM Deluxe cervical goniometer	Neck flexion increased significantly in groups 1 [5.45°] and 3 [7.30°] and neck extension increased significantly in all groups [group 1: 6.10°/ group 2: 3.55°/ group 3: 6.65°]. Right lateral flexion increased significantly in all groups in all groups [group 1: 7.95°/ group 2: 5.00°/ group 3: 7.50°]. Lateral flexion left [group 1: 7.00°/ group 2: 1.95°/ group 3: 6.60°] increased statistically in all groups as well rotation to both sides increased statistically significant right [group 1: 7.25°/ group 2: 3.15°/ group 3: 9.05°], left [group 1: 6.85°/ group 2: 2.50°/ group 3: 7.55°]. In groups 1 and 3, all movements increased above the minimal detectable difference [MDC] > 5°. In group 2 only lateral flexion right reached [MDC] >5°] Effect sizes between group 2 and group 3: Flexion: 0.54 Extension: 0.26 Lateral Flexion right: 0.38 Lateral flexion left: 0.64 Rotation right: 0.66 Rotation left: 0.67	Some concerns
Arjona et al. [2021]	96 participants [both sexes] Chronic [at least 3 months] mechanical neck pain 18-40 years [Mean age 29.5 ± 5.2 years] Group 1 [n = 31]: mean age: 28.71 ± 5.53 Group 2 [n = 31: mean age 29.33 ± 4.80 Group 3 [n = 34: mean age: 30.29 ± 5.14	Upper Cervical spine	Group 2: INYBI group [Instrumental suboccipital inhibition] Group 3: INYBI and upper cervical manipulation technique Two sessions one week apart	Group 1: Manual inhibition]	Immediately post-treatment Pre-treatment and immediately post-treatment [One-week post-treatment]	Cervical range of motion [CROM device] every plane	No statistically significant between group differences. Within group 2 there were three time points at which the recent measurements were statistically significantly different compared to the baseline treatment. Right rotation left rotation after the first intervention [62.63° to 65.33°/ 62.60 ° to 65.43°], and right side bending after the second intervention [38.03° to 42.03°]. Within group 3 there were measurements statistically significant for Flexion baseline [55.73°] to after second intervention [60.12°], Extension pre-intervention of second week [63.65 °] to after the second intervention [66.15°], rotation right baseline [62.71°] to after second intervention [67.73°], rotation left baseline [64.85°] to after first intervention [67.53°] and after the second intervention [71.47°], right side bending between pre-intervention of second week [37.94°] and after the second intervention [40.59°], left side bending to baseline [44.23°] and pre-intervention second week [44.85°] and after the second intervention [48.24°]. Effect sizes between group 2 and 3	Some concerns
							Flexion: Pre to Post1: 0.08, Pos1 to Post2: 0.11 Post2 to Post3: 0.09. Pre to Post3: 0.2 Extension: Pre to Post1: 0.08, Post1 to Post2: 0.11 Post2 to Post3: 0.09. Pre to Post3: 0.7 Right rotation: Pre to Post1: 0.05, Post1 to post2: 0.17, Post2 to Post3: 0.25. Pre to Post3: 0.23 Left rotation: Pre to Post1: 0.01, Post1 to Post2: 0.13 Post2 to Post3: 0.24. Pre to Post3: 0.35 Right side bending: Pre to Post1: 0.03, Post1 to Post2: 0.45 Post2 to Post3: 0.23. Pre to Post3: −0.16 Left side bending Pre to Post1: 0.21, Post1 to Post2: 0.38 Post2 to Post3: 0.30, Pre to Post3: 0.11 MDC was not assessed.	
Brück et al. [2021]	60 participants [both sexes]. Chronic neck pain [> 3 months]> NRS 2 30-65 years MT [n = 20: mean age 50.1 ± 11.8] CG [n = 20: mean age 45.7 ± 9.4]	Cervical and upper thoracic	Two sessions of high- velocity low-amplitude [HVLA] manipulation for each restricted segment [cervical: rotation technique, thoracic: drop technique	2] No treatment 3] Fascial treatment [2 sessions]	After the last treatment session	Cervical range of motion of all planes [CROM device]	ROM increased for all directions in the fascial treatment group by at least 8.9° and in the HVLA group by at least 4.8°. The control group remained unchanged. Significant group*time interaction for all directions; repeated measure t-tests: large ES for fascial treatment, medium to large ES for HVLA. Improvement of range of motion in the HVLA group compared to the control group. No correction for multiple testing. No MDC was reported. Effect sizes [between group1 and group 2]: Right rotation: −0.72, left rotation −0.88, Side bending right:0.46, Side bending left:0.90, Extension:0.87 Flexion:0.81	Low
Cassidy et al. [1992]	Patients with unilateral neck pain with referral to the trapezius HVLA group [n = 52: mean age 34.5 ± 13.0 Mobilization group [n = 48: mean age 37.7 ± 12.5	Cervical	HVLA to cervical spine [1 thrust] 1 session	Mobilization to cervical spine [muscle energy technique] 1 session	Immediately post-treatment [within 5 minutes]	Active cervical ROM [flexion, extension, ipsilateral and contralateral lateral flexion, ipsilateral and contralateral rotation] Goniometer [CROM Device]	Flexion increased by 5.1 ° in the manipulation group and by 3.9° in the mobilization group. Extension increased by 3.1° in the manipulation group and by 1.3 ° in the mobilization group. Ipsilateral rotation increased by 5.0 ° in the manipulation group and by 4.2° in the mobilization group. Contralateral rotation increased by 3.6° in the manipulation group and by 2.4° in the mobilization group. Ipsilateral flexion increased by 3.4 ° in the manipulation group and by 2.0° in the mobilization group. Contralateral flexion increased by 4.3 ° in the manipulation group and by 3.0 ° in the mobilization group. There was no statistically significant difference between the change score of the manipulation and the mobilization group [p-values between 0.25 and 0.67]. Effect sizes between groups: Flexion: 0.18 Extension: 0.17 Ipsilateral Rotation: 0.06 Contralateral Rotation: 0.11 Ipsilateral Flexion: 0.14 Contralateral Flexion: 0.15	Some concern
Espí-López et al. [2016]	Subjects with tension-type headache [TTH] frequent or chronic HVLA group [n = 51: mean age 37.7 ± 10.6 Control group [n = 51: mean age 40.5 ± 11.3	Cervical	HVLA to cervical spine [C0/C1, each side] AND Massage to the cervical spine [10 minutes, focus cervical/occipital muscles] AND 10 min rest [As in control group] 4 sessions over 4 weeks	Massage to cervical spine [10 minutes, focus cervical/occipital muscles] AND 10 min rest 4 sessions over 4 weeks	Post-treatment [8 weeks post-treatment]	Active cervical ROM [upper cervical and cervical flexion, extension] CROM device	Upper cervical spine flexion changed from 8.31° [pre-test] to 11.16° [post-test] to 11.06 ° [follow-up] in the massage group. Upper cervical spine extension changed from 15.00° [pre-test] to 18.80 [post-test] to 18.96° [follow-up] in the massage group. Upper cervical spine flexion changed from 7.82° [pre-test] to 13.16° [post-test] to 11.67 ° [follow-up] in the massage + manipulation group. Upper cervical spine extension changed from 16.22° [pre-test] to 23.25 [post-test] to 22.20° [follow-up] in the massage + manipulation group. Cervical spine flexion changed from 48.18° [pre-test] to 55.22° [post-test] to 53.88 ° [follow-up] in the massage group. Cervical spine extension changed from 45.53° [pre-test] to 51.37[post-test] to 51.31 [follow-up] in the massage group. Cervical spine flexion changed from 52.57° [pre-test] to 54.31° [post-test] to 52.59 ° [follow-up] in the massage + manipulation group. Cervical spine extension changed from 46.12° [pre-test] to 52.27 [post-test] to 50.73° [follow-up] in the massage + manipulation group. Interaction group * time point significant for MANOVA; post-hoc tests sig. [p`s < 0.05] for two of the four directions tested [sig for flexion and upper cervical flexion, with small to medium effect sizes. Not significant for extension and upper cervical extension]. Effect sizes between groups: Upper cervical flexion: −0.59 Upper cervical extension: −0.37 Cervical flexion: 0.48 Cervical extension: −0.02 No MDC was reported in the study.	Low risk
Galindez-Ibarbengoetxea et al. [2017]	Asymptomatic volunteers Indiscriminate HVLA group [AMC5] [n = 12: mean age 30.5 ± 3.1] HVLA group [MT] [n = 12: mean age 30.8 ± 4.0] Sham group [ST] [n = 12: mean age 31.2 ± 2.2]	Cervical OR Cervical or thoracic	Group 1] Indiscriminate HVLA group: HVLA to C5 right OR Group 2] HVLA group [MT]: HVLA to cervical or upper thoracic spine [based on diagnosed dysfunction] 1 session	Group 3] Sham group [ST]: Same positioning indiscriminate HVLA group, no thrust,3 rotation movements 1 session	Immediately post-treatment	Active cervical ROM [flexion, extension, right/left lateral flexion, right/left rotation] CROM goniometer	Within group change: Flexion AMC5: 4.30° MT: 5.16° ST:1.59° Extension AMC5: 6.98° MT: 10.44° ST: −3.37° Right side bending AMC5: 3.62 MT: 7.65 ST: 5.98 Left side bending: AMC5: 6.90° MT: 10.05° ST: 5.85° Right rotation: AMC5: 8.70° MT: 7.66° ST: 7.09° Left rotation: AMC5: 4.03 MT: 12.25° ST: 5.81° AMC 5 vs ST or MT vs ST meaningful comparisons Significant group interaction [p < 0.01]. The between group difference in change for left rotation between AMC5 and ST, MT and ST. No MDC was reported Effect sizes between groups: Flexion: Group 1 vs Group 2: −0.05 Group 1 vs Group 3: 0.22 Group 2 vs Group 3: 0.27 Extension: Group 1 vs Group 2: −0.85 Group 1 vs Group 3: 0.64 Group 2 vs Group 3: 1.49	Low risk
							Right side bending: Group 1 vs Group 2: −0.27 Group 1 vs Group 3: −0.08 Group 2 vs Group 3: 0.19 Left side bending: Group 1 vs Group 2: 0.05 Group 1 vs Group 3: −0.27 Group 2 vs Group 3: −0.32 Right rotation: Group 1 vs Group 2: 0.06 Group 1 vs Group 3: 0.00 Group 2 vs Group 3: −0.06 Left rotation: Group 1 vs Group 2: −0.52 Group 1 vs Group 3: −0.08 Group 2 vs Group 3: 0.44	
Gomez et al. [2020]	44 patients [both sexes] Chronic [chronic >3 months] non-specific neck pain higher than 2 on the 11 numeric pain rating scale and positive cervical flexion rotation test on one side. 18-60 years Experimental group [n = 22: mean age 38.36 ± 11.66] Control group [n = 22: mean age 37.50 ± 11.83]	Upper cervical spine	The experimental group received a single upper cervical spine rotational thrust To the side of the positive CFRT 1 Session	Sham technique [Physiotherapist places his hands on the patients’ neck and turns off ultrasound] 1 Session	Immediately post-treatment [seven and fifteen days post-treatment]	Cervical flexion rotation test with CROM instrument [goniometer]	Rotation differed between groups at baseline [1.22 °], post-immediate [8.31°], after seven days [7.90°], and after 15 days [6.81°]. The experimental group showed an increase of limitation rotation post-immediate of 7.4°, seven days 6, 8° and 15 days 6.13°. MDC reported 7° degrees are considered meaningful [Hall et al. 2004] Effect size between experimental group over time compared with control group as calculated by Gomez et al. [2020]: 0.75	Some concerns
Gorrell et al. [2016]	Volunteers with mechanical neck pain > 1 month HVLA group [n = 21: mean age 24.4 ± 4.0] Instrument group [IAM] [n = 22: mean age 25.0 ± 4.9] Stretching group [n = 22: mean age 23.8 ± 3.5]	Cervical	HVLA to the cervical spine [1 thrust] AND active cervical muscle stretching routine 1 session OR IAM to the cervical spine [1 application] AND active cervical muscle stretching routine 1 session	Active cervical muscle stretching routine 1 session	Immediately post-treatment	Active cervical ROM [flexion, extension, left/right lateral flexion, left/right rotation] Digital dual inclinometer	Immediate change in rotation bilaterally [Ipsilateral [10.35°]: F = 11.17, p = .002; contralateral [6.32°]: = 6.44, p = .015] for the HVLA group. There was a between group difference for lateral flexion on the contralateral side to manipulation [F = 12.44, p = .001] in HVLA [6.40°] vs. IAM. No significant differences were found in other directions. Statistics for comparison to the control group [only stretching] were not reported. No MDC was reported Effect sizes could not be calculated as baseline data for ROM was not published.	Some concerns
Lopez-Lopez et al. [2015]	Patients with chronic neck pain > 3 months HVLA group [n = 15: mean age 35.4 ± 8.0] Mobilization group [n = 16: mean age 36.0 ± 9.9] SNAG group [n = 17: mean age 37.8 ± 8.6]	Cervical	HVLA to the cervical spine [one side, maximum 2 thrusts] 1 session	Mobilization to the cervical spine [one side, grade III PA, 3x 2 minutes] 1 session OR Sustain apophyseal natural glide [SNAG, 10x3 series] 1 session	5 min post-treatment	Active cervical ROM [flexion/extension, lateral flexion, rotation] CROM device	Rotation changed preintervention – post-intervention in the HVLA group [11.63°], mobilization group [5.14], and SNAG group [4.45°]. Lateral flexion changed preintervention – postintervention in the HVLA group [7.95°], mobilization group [4.78°], and SNAG group [7.35°]. Flexion-extension changed pre-intervention – post-intervention in the HVLA group [37.14°], mobilization group [17.68°], and SNAG group [18.69°]. Trend for the three-way treatment x anxiety x time interaction with respect to CROM in lateral flexion: Mobilization better under high anxiety condition, SMT under low anxiety condition. MDC was reported. Effect sizes between groups: CROM rotation: HVLA group vs mobilization group: −0.77 HVLA group vs SNAG group: 0.53 Mobilization group vs SNAG group: 1.30 CROM lateral-flexion: HVLA group vs mobilization group: −3.36 HVLA group vs SNAG group: −0.25 AP mob vs SNAG: 3.11 CROM flexion-extension: HVLA group vs mobilization group: 1.54 HVLA group vs SNAG group: 1.54 Mobilization group vs SNAG group: 0.0	Low risk
Martínez-Segura et al. [2006]	Patients with mechanical neck pain ≥ 1 month HVLA group [n = 34: mean age 35 ± 10] Control group [n = 37: mean age 39 ± 10]	Cervical	HVLA to cervical spine [C3/4 or C4/5, 1 thrust] 1 session	Mobilization to the cervical spine [no thrust, position held 30 seconds] 1 session	5 minutes post-treatment	Active cervical ROM [flexion, extension, right/left lateral flexion, right/left rotation] CROM Device	Cervical flexion improved by 7° in the HVLA group and 1.5 ° in the mobilization group. Cervical extension improved by 8° in the experimental group and 1.4 ° in the mobilization group. Left lateral flexion improved by 5 ° in the HVLA group and 0.8° in the mobilization group. Right lateral flexion improved by 5 ° in the experimental group and 0.8° in the control group. Left rotation improved by 9 ° in the experimental group and 0.3° in the mobilization group. Right rotation improved by 10° in the experimental group and 0.4° in the mobilization group. The intergroup comparison [unpaired t-test analysis] between the improvement [pre-post scores] in both groups showed that the experimental group obtained a greater improvement than the mobilization group in all the outcome measures [P < .001]. Pearson correlation test showed a negative association between the improvement in neck pain at rest and the improvement in each cervical range of motion [all P < .001]. No MDC was reported Effect sizes between groups: Cervical flexion: 0.89 Cervical extension: 0.76 Left lateral flexion: 0.68 Right lateral flexion: 0.71 Left rotation: 0.95 Right rotation:1.11	Some concerns
Ouseley and Parkin-Smith [2002]	Patients with chronic tension-type headache frequency of at least 15 days per month HVLA group [n = 5: average age 32] Control group [n = 6: average age 48]	Cervical	HVLA to the cervical spine Max. 8 sessions over 4 weeks	Mobilization to the cervical spine Max. 8 sessions over 4 weeks	end of each treatment week comparison between baseline and final treatment recordings	Cervical ROM [flexion, extension, left/right lateral flexion, left/right rotation] Cervical inclinometer	Within the HVLA group, the following changes [pre-post treatment] were reported: Flexion: −6° Extension: – 0.4° Right rotation: 1.5° Left rotation: 10.4 ° Right lateral flexion: – 1.4° Left lateral flexion: 0.3° For the mobilization group, the following changes [pre-post treatment] were reported: Flexion: −1.3° Extension: 5.1° Right rotation: 6.5° Left rotation: 5.4° Right lateral flexion: 1.3° Left lateral flexion: −3.6° No significant intra- or intergroup changes in ROM between baseline and final treatment. No statistical comparison of pre-post differences between groups. No MDC was reported. Effect sizes between groups: Flexion: −0.13 Extension: −0.49 Rotation right: −0.36 Rotation left: 0.29 Lateral flexion right: −0.42 Lateral flexion left: 0.84	Some concerns
Reynolds et al. [2020]	Patients with temporomandibular disorder HVLA group [n = 25: mean age 32.2 ± 11.3] Sham group [n = 25: mean age 38.8 ± 14.8]	Cervical	HVLA to C0/C1 and C1/2 [max 2 thrusts each side = 4–8 thrusts] AND 2 minutes suboccipital release, education, and exercise. 4 sessions over 4 weeks.	Sham to C0/C1 and C1/2 [= 4 shams, same position for 15 seconds, no thrust] AND 2 minutes suboccipital release, education, and exercise. 4 sessions over 4 weeks.	1 week after baseline [visit 2] End of treatment [visit 4, week 4]	Cervical ROM [seated flexion, extension, supine rotation] inclinometer	There was an immediate change of ROM for cervical flexion of 1.44° in the HVLA group and 1.36° in the sham group, between baseline and one week 0.96° for the HVLA group, and 0° in the sham group and baseline and four weeks 5.12° in the HVLA group and 4.8° in the sham group. None was statistically significant. There was an immediate change of ROM for cervical extension of 6.72° in the HVLA group and 4.92° in the sham group, between baseline and one week 4.24° for the HVLA group, and 3.04° in the sham group and baseline and four weeks 7.84° in the HVLA group and 3.52° in the sham group. None were statistically significant. There was an immediate change of ROM for right cervical rotation of 2.2° in the HVLA group and 1.72° in the sham group, between baseline and one week 2.92° for the HVLA group and 2.88° in the sham group and baseline and four weeks 7.84° in the HVLA group and 4.44° in the sham group. None were statistically significant. There was an immediate change of ROM for left cervical flexion rotation of 5.8° in the HVLA group and 1.44° in the sham group, between baseline and one week 6.24° for the HVLA group and – 0.16° in the sham group and baseline and four weeks 8.08° in the HVLA group and 3.44° in the sham group. None was statistically significant. No MDC was reported for cervical spine ROM	Some concerns
							Effect sizes between groups: Flexion: Immediately: 0.01 1 week: 0.08 4 weeks: 0.03 Extension: Immediately: 0.15 1 week: 0.09 4 weeks: 0.31 Rotation right: Immediately: 0.07 1 week: 0.02 4 weeks: 0.32 Rotation left: Immediately: 0.54 1 week: 0.69 4 weeks: 0.62	
Valera-Calero et al. [2019]	Patients with mechanical neck pain of at least 3 months duration HVLA group [n = 28] 35.6 ± 8.1 Mobilization group [n = 28: mean age 37.3 ± 10.5] Sham group [n = 27: Mean age 37 ± 8.9]	Cervical	HVLA to cervical spine [1 thrust] 1 session	Mobilization to the cervical spine [3x 1 minute] 1 session OR Sham manipulation to the cervical spine [same position as HVLA, no thrust, drop piece, as described by Vernon et al] 1 session	Immediately post-treatment 1 week posttreatment	Active cervical ROM [Flexion, extension, left and right lateral flexion, left and right rotation] CROM device	Left rotation changed in the cervical manipulation group from pre-treatment [66.25°] to post-treatment [74.36°] 7.84° and from post- treatment [74.36°] to 1-week follow-up [71.54°] – 2.82 °. In the cervical mobilization group left rotation changed from pre-treatment [63.36°] to post-treatment [69.39°] 6.03° and from post-treatment [69.39°] to 1-week follow-up [65.11°] – 4.28°. In the sham group pre-treatment [62.52°] to post treatment [68.11°] 5.59° and from post treatment [68.11°] to 1-week follow-up [68.78°] – 0.67°. Right rotation changed in the cervical manipulation group from pre-treatment [61.86°] to post treatment [72.29°] 10.43° and from post treatment [72.29°] to 1-week follow-up [70.93°] – 1.36 °. In the cervical mobilization group right rotation changed from pre-treatment [65.00°] to post-treatment [68.14°] 3.14° and from post-treatment [68.14°] to 1-week follow-up [66.57°] – 1.57°. In the sham group pre-treatment [64.26 °] to post-treatment [68.74°] 4.48° and from post treatment [68.74°] to 1-week follow-up [71.70°] 2.96°.	Low risk
							Extension changed in the cervical manipulation group from pre-treatment [55.14°] to post-treatment [58.54°] 3.4° and from post-treatment [58.54°] to 1-week follow-up [57.86°] – 0.68°. In the cervical mobilization group, extension changed from pre-treatment [54.07°] to post-treatment [55.79°] 1.72° and from post-treatment [55.79°] to 1-week follow-up [56.21°] 0.42°. In the sham group pre-treatment [57.15 °] to post-treatment [53.11°] −4.04° and from post-treatment [53.11°] to 1-week follow-up [54.44°] 1.33°. Flexion changed in the cervical manipulation group from pre-treatment [59.68°] to post-treatment [64.82°] 5.14° and from post-treatment [64.82°] to 1-week follow-up [62.61°] – 2.21°. In the cervical mobilization group flexion changed from pre-treatment [54.75°] to post-treatment [56.46°] 1.71° and from post-treatment [56.46°] to 1-week follow-up [51.89°] – 4.57°. In the sham group pre-treatment [57.52 °] to post-treatment [56.15°] −4.04° and from post treatment [56.15°] to 1-week follow-up [56.67°] 0.52°.	
							Left lateral flexion changed in the cervical manipulation group from pre-treatment [37.82°] to post-treatment [38.71°] 0.89° and from post-treatment [38.71°] to 1-week follow-up [39.14°] 0.43°. In the cervical mobilization group, left lateral flexion changed from pre-treatment [39.57°] to post-treatment [41.29°] 1.72° and from post-treatment [41.29°] to 1-week follow-up [40.25°] – 1.04°. In the sham group, pre-treatment [38.00°] to post-treatment [37.89°] −0.11° and from post-treatment [37.89 °] to 1-week follow-up [34.74°] – 3.42°. Right lateral flexion changed in the cervical manipulation group from pre-treatment [40.11°] to post-treatment [39.11°] – 1.0° and from post-treatment [39.11°] to 1-week follow-up [40.11°] 1.0°. In the cervical mobilization group, left lateral flexion changed from pre-treatment [37.21°] to post-treatment [37.21°] 0° and from post-treatment [37.21°] to 1-week follow-up [36.46°] – 0.75°. In the sham group, pre-treatment [37.96°] to post-treatment [36.44°] −1.52° and from post-treatment [36.44 °] to 1-week follow-up [35.33°] – 1.11°. Significant interaction effect for flexion [p = 0.015] and right rotation [P = 0.001] with medium effect sizes.	
							Significant increase in flexion immediately post-treatment in the HVLA group, significantly greater vs cervical mobilization and sham [P = 0.001] and in HVLA vs sham at one week follow up [P = 0.001]. Significant increase in right rotation immediately post-treatment in HVLA [P < 0.001] and sham manipulation [P = 0.009], improvement in right rotation reversed at one week for cervical HVLA [P < 0.001], remained for sham [P = 0.001]. No MDC was reported. Effect sizes between groups: Left rotation: Immediately: Cervical man vs cervical mob: 1.09 Cervical man vs sham: 1.32 Cervical mob vs sham: 0.23 1 week: Cervical man vs cervical mob: 2.03 Cervical man vs sham: −0.42 Cervical mob vs sham: −2.45 Right rotation: Immediately: Cervical man vs cervical mob: 3.98 Cervical man vs sham: 3.25 Cervical mob vs sham: −0.73 1 week: Cervical man vs cervical mob: 4.90 Cervical man vs sham: 1.07 Cervical mob vs sham: −3.84 Extension Immediately: Cervical man vs cervical mob: 0.66 Cervical man vs sham: 1.86 Cervical mob vs sham: 1.20 1 week:	
							Cervical man vs cervical mob: 0.23 Cervical man vs sham: 2.13 Cervical mob vs sham: 1.90 Flexion Immediately: Cervical man vs cervical mob: 2.20 Cervical man vs sham: 4.18 Cervical mob vs sham: 1.98 1 week: Cervical man vs cervical mob: 3.71 Cervical man vs sham: 2.42 Cervical mob vs sham: −1.29 Left lateral flexion Immediately: Cervical man vs cervical mob: −0.59 Cervical man vs sham: 0.70 Cervical mob vs sham: 1.29 1week Cervical man vs cervical mob: 0.40 Cervical man vs sham: 2.91 Cervical mob vs sham: 2.52 Right lateral flexion: Immediately: Cervical man vs cervical mob: −0.68 Cervical man vs sham: 0.58 Cervical mob vs sham: 1.25 1 Week: Cervical man vs cervical mob: 0.50 Cervical man vs sham: 1.74 Cervical mob vs sham: 1.24	
Vernon et al. [2012]	Patients with mechanical neck pain > 3 months HVLA group [n = 32: mean age 38.3 ± 9.9] Sham group [n = 32: mean age 38.8 ± 11.3]	Cervical	HVLA to cervical spine [each side] 1 session	Sham to cervical spine [each side, same positioning, no thrust, use of drop to mimic sound] 1 session	5 and 15 mins posttreatment	Cervical ROM [all 3 cardinal planes]	ROM measures not changed pre-post treatment, no interaction with group [p-value not given, no statistics shown]. No effect sizes could be calculated due to lack of detailed reporting of ROM data. No MDC for ROM was reported.	Low risk
Whittingham and Nilsson [2001]	Patients with cervicogenic headache > 6 months Group 1 [n = 49: mean age 39.4 ± 11.6 yrs] [sham, HVLA, washout] Group 2 [n = 56 mean age 41.9 ± 12.5] [HVLA, washout, sham]	Cervical	HVLA to the upper cervical spine [single thrust, toggle recoil manipulation] 3 times/week	Sham to the cervical spine [single sham, deactivated Pettibon instrument] 3 times/week	At the end of each phase, baseline obs. weeks 3, 6, 9, 12 , at the end of the intervention phase	Active cervical ROM [left/right-lateral flexion, left/right rotation] Goniometer [inclinometer]	In the manipulation group, right rotation changed from 57° at baseline over 57° [week 3], 67° [week 6], 69° [week 9] to 70° [week 12]. Left rotation changed from 54° at baseline over 55° [week 3], 67° [week 6], 68° [week 9] to 69° [week 12]. In the manipulation group, right-lateral flexion changed from 38° at baseline over 37° [week 3], 46° [week 6], 46° [week 9] to 47° [week 12]. In the manipulation group, left lateral flexion changed from 36° at baseline over 36° [week 3], 44° [week 6], 44° [week 9] to 45° [week 12]. In the sham group, right rotation changed from 56° at baseline over 56° [week 3], 57° [week 6], 73° [week 9] to 73° [week 12]. Left rotation changed from 54° at baseline over 54° [week 3], 56° [week 6], 71° [week 9] to 72° [week 12]. In the manipulation group, right-lateral flexion changed from 39° at baseline over 39° [week 3], 39° [week 6], 48° [week 9] to 40° [week 12]. In the manipulation group, left lateral flexion changed from 38° at baseline over 38° [week 3], 39° [week 6], 47° [week 9] to 47° [week 12]. All four ROM measures significantly [p`s < 0.006] improved by treatment; no change by sham treatment. No direct statistical comparison between groups [I.e., no time x group interaction effect] was reported. Strength: The cross-over design shows similar behavior in both groups. No MDC was reported.	Low risk
							Effect sizes between groups: Right rotation: Week 0: −0.69 Week 3: −0.67 Week 6: −7.67 Week 9: 2.91 Week 12: 2.49 Left rotation: Week 0: 0.0 Week 3: −0.67 Week 6: −8.44 Week 9: 2.19 Week 12: 2.19 Right lateral flexion: Week 0: 0.79 Week 3: 2.22 Week 6: −4.34 Week 9: 1.60 Week 12: −5.56 Left lateral flexion: Week 0: 1.60 Week 3: 1.59 Week 6: −4.00 Week 9: 2.30 Week 12: 1.82	
**Thoracic and lumbar spine**								
Kardouni et al. [2015]	Patients with subacromial impingement syndrome > 6 weeks HVLA group [n = 26: mean age 30.8 ± 11.9] Sham group [n = 26: mean age 33.2 ± 12.6]	Thoracic	HVLA manipulation at the upper, middle, and lower thoracic spine 1 session, each region 2x [= 6 thrusts total]	Sham intervention [identical positioning, minimal pressure] at the upper, middle, and lower thoracic spine 1 session, each region 2x [= 6 sham interventions total]	posttreatment	Active thoracic excursion [full flexion to full extension] Electromagnetic motion capture system	Thoracic extension during arm elevation in the HVLA group changed from pre-treatment to post-treatment as follows: 30°: 0,6 to 0.1 [−0.5] 60°: 0.8 to 0.4 [−0.4] 90°: 1.0 to 0.7 [−0.4] 120 °: 2.4 to 2.2 [−0.2] Thoracic extension during arm elevation in the sham group changed from pre-treatment to post-treatment as follows: 120°: 1.6 to 1.6 [0] 90°: 1.8 to 1.8 [0] 60°: 2.3 to 1.9 [−0.4] 30°: 4.4 to 4.1 [−0.3]	Low risk
							Thoracic extension during arm lowering in the HVLA group changed from pretreatment to post-treatment as follows: 30°: 4.6 to 4.4 [−0.2] 60°: 2.9 to 2.5 [−0.4] 90°: 1.8 to 1.4 [−0.4] 120°: 1.5 to 1.0 [−0.5] Thoracic extension during arm lowering in the sham group changed from pretreatment to post-treatment as follows: 120°: 16.4 to 5.9 [−0.5] 90°: 4.4 to 4.2 [−0.1] 60°: 3.1 to 3.1 [0] 30°: 2.4 to 2.4 [0] MDC was reported for thoracic extension [1.4° - 2.1°] Thoracic extension in the HVLA group changed from 37.2° pre-treatment to 37.7° post-treatment, and in the sham group from 30.07 to 31.7 °. No significant difference between the groups in thoracic extension. Effect Sizes between groups: Rest: 0.20 Ascending 30°: 0.25 Ascending 60°: 0.24 Ascending 90°: 0.20 Ascending 120°: 0.32 Descending 30°: 0.28 Descending 60°: 0.33 Descending 90°: 0.32 Descending 120°: 0.25	
Ditcharles et al. [2017]	Right-handed young healthy adults HVLA group [n = 11: mean age 28 ± 4] sham group [n = 11: mean age 29 ± 4]	Thoracic	HVLA to T9 1 session	Sham intervention [light touch only, same position for 10 seconds] 1 session	Immediately post-treatment	Active thoracic and lumbar ROM [thoracic flexion/extension and lumbar flexion/extension] 2 inclinometers	A significant difference in thoracic flexion [main effect of GROUP[F = 4.53, p < 0.05], CONDITION [F = 15.73, p < 0.01] and GROUP – CONDITION interaction [F = 14.55, p < 0.01]. Post-hoc: Significant improvement in the SMT group, no change in the control group. No significant effect on any of the other ROM values. No effect sizes could be calculated due to lack of detailed reporting of ROM data. No MDC was reported.	Low risk
Nambi et al. [2018]	Patients with untreated uncomplicated chronic low back pain, lumbago, sciatica [above knee], with pain localized in L4/L5 HVLA group [n = 110] Laser group [n = 110] Control group [n = 110] Age: > 18 years, no mean given	Lumbar	HVLA to L4/5 [maximum of 2 thrusts on each side] AND laser therapy AND conventional exercises 3 sessions/week for 4 weeks	Laser therapy AND Conventional exercises OR Conventional exercises 3 sessions/week for 4 weeks	4 weeks [end of treatment duration], 6 months, 12 months	Active lumbar ROM [flexion by modified Schober test]	The modified Schober changed in the HVLA group from 17.39° at baseline to 18.67° [four weeks], 19.17° [6 months], 20.22° [12 months]. In the therapy group, the modified Schober changed from 17.25° at baseline to 18.10° [four weeks], 18.67° [6 months], and 19.48° [12 months]. The time and group interaction for modified Schober was significant [p < 0.05]. No MDC was reported. Effect sizes between groups Baseline: −0.24 4 weeks: −1.23 6 months: −0.55 12 months: −1.08	Some concern
Passmore et al. [2019]	Patients with degenerative lumbar spinal stenosis HVLA group [n = 7: mean age 59.1 ± 9.3] Control group [n = 7: mean age 58.9 ± 12.6]	Lumbar	HVLA to the lumbar spine [bilateral, hypothenar lumbar pull, side posture] 1 session	Waiting 5 minutes 1 session	Post-treatment	Active lumbar ROM [flexion, extension, left/right lateral flexion, left/right rotation] Electrogoniometry	Flexion in the HVLA group changed from pre-HVLA 20.09° to post-HVLA 24.7° and in the sham group from 26.2° to 22.09°. Extension in the HVLA group changed from pre-HVLA 19.4° to post-HVLA 21.9° and in the sham group from 23.3° to 20.7°. Left lateral flexion in the HVLA group changed from pre-HVLA 16.3° to post-HVLA 20.4°, and in the sham group from 18.9° to 21°. Right lateral flexion in the HVLA group changed from pre-HVLA 15.9° to post-HVLA 18.7° and in the sham group from 17.9° to 20.9°. Left rotation in the HVLA group changed from pre-HVLA 11.3° to post-HVLA 25.7° and in the sham group from 11.7° to 10.7°. Right rotation in the HVLA group changed from pre-HVLA 15.7° to post-HVLA 13.4°, and in the sham group from 10.4° to 10.3°. No difference within or between groups in lumbar ROM posttreatment based upon exploratory analysis. No MDC was reported Effect sizes between groups: Flexion: −0.16 Extension: −0.12 Left lateral flexion: 0.08 Right lateral flexion: 0.25 Left rotation: −0.65 Right rotation: −0.29	Some concern

**Table 2:** Regional effects of HVLA thrust on range of motion.

Autor, Year	Participants [A]Symptomatic Age Number	Treatment region/ Region of interest	Intervention	Control	Time to assessment [relative to intervention]	Biomechanical outcome parameters	Main biomechanical Results	RoB Rating
Erdem et al. [2021]	80 male participants with chronic mechanical [> 3 months] neck pain With or without arm pain 18-25 years Manipulation group [n = 50: median age 20] Control group [n = 30: median age: 20.5]	Thoracic spine	Single session of thoracic manipulation	Rest for 5 minutes	Immediately post-intervention	Active cervical flexion, extension, lateral flexion, and rotation [CROM device]	Range of motion changed from pre to post-intervention in the HVLA group for flexion 2 °, for Extension 2° for left lateral flexion 2 °, for right lateral flexion 2° for left rotation 2° and for right rotation 2°. For the control group, flexion changed 0°, extension 2°, left lateral flexion 2°, right lateral flexion 3.5°, left rotation 2° and right rotation 2°. A statistically significant difference between groups for flexion and right rotation. No effect sizes could be calculated due to lack of detailed reporting of ROM data. No MDC was reported.	Some concerns
González-Iglesias et al. [2009a]	Patients with acute mechanical neck pain < 1 month HVLA group [n = 23: mean age 34 ± 5] Control group [n = 22: mean age 34 ± 6]	Thoracic	HVLA to thoracic spine [maximum 2 thrusts] 1x/week for 3 weeks AND Superficial thermo- and electro-therapy and soft tissue massage 2x/week for 3 weeks	Superficial thermo- and electro-therapy and soft tissue massage 2x/week for 3 weeks	1-week post-treatment	Active cervical ROM [flexion, extension, right/left lateral flexion, right/left rotation] CROM Device	In the control group, cervical flexion changed 0.9° from 44.7° to 45.6°. Cervical extension changed by 1.3°, from 58.8° to 60.1°. Left lateral- flexion changed by 1.4°, from 40.2° to 41.6°. Right lateral-flexion changed by 1.6°, from 39.4° to 41.0°. Left rotation changed by 0.6°, from 57.8° to 58.4° and right rotation changed by 0.2°, from 56.1° to 56.3°. In the HVLA group cervical flexion changed by 11.6°, from 45.6° to 57.2°. Cervical extension changed by 11.3°, from 59.1° to 70.3°. Left lateral- flexion changed by 9.4°, from 39.1° to 48.5°, right lateral-flexion changed by 11°, from 36.2° to 47.2. Left rotation changed by 9°, from 59.2° to 68.2°. Finally, right rotation changed by 9.8°, from 55.8.1° to 65.6°. There was greater improvement in all ROM measures for the treatment group compared to the control group [all p`s < 0.001] with large effect sizes. Effect Sizes between Groups: Cervical flexion: 2.07 Cervical extension: 1.46 Left lateral flexion: 1.39 Right lateral flexion: 1.25 Left rotation: 1.71 Right rotation: 1.34 No MDC was reported for CROM.	Low risk
Krauss et al. [2008]	Patients with cervical pain HVLA group [n = 22: mean age 35.0 ± 10.5] Control group [n = 10: Mean age 34.2 ± 9.6]	Thoracic	HVLA to upper thoracic spine [T1-T4] 1 session	No intervention, seated for equal time	Immediately posttreatment	Active cervical ROM [left/right rotation] CROM	Within group changes of left rotation in the control group were – 0.6° and 7.09° in the HVLA group. For right rotation, it was −0.1° in the control group and 8.23° in the HVLA group. Statistically significant GROUP x TIME interaction for right rotation and left rotation [p < 0.05 in favor of SMT. No effect sizes could be calculated due to lack of detailed reporting of ROM data. MDC reported for right and left rotation.	Low risk
Suvarnnato et al [2013]	Patients with chronic mechanical neck pain > 3 months HVLA group: [n = 13: mean age 37.0 ± 12.5] Mobilization group [n = 13: mean age 39.9 ± 11.5] Control group [n = 13: mean age 35.3 ± 11.0]	Thoracic	HVLA to thoracic spine [T6/7, maximum of 2 thrusts] 1 session	Mobilization group: Single-level thoracic mobilization to T6/7 [both sides, grade III, 1 minute] OR Control group: Prone position, clinician hands on T6/7 for 2 minutes 1 session	Immediate posttreatment 24 hours posttreatment	Active cervical ROM [flexion/extension, left/right lateral flexion, left/right rotation] CROM device	In the control group cervical flexion changed −1.95° from 60.57° to 58.62° from baseline to immediately after treatment and −0.26° from immediately after treatment to 24 hours follow-up. Cervical extension changed by 2.31°, from 47.64 to 49.95 from baseline to immediately after treatment and by −0.67° from 49.95° to 49.28° from immediately after treatment to 24 hours follow-up. Left lateral flexion changed by 1.81°, from 33.03° to 34.84° from baseline to immediately after treatment and – 0.74 °, from 34.84 to 34.10° from immediate after treatment to 24 hours follow-up. Right lateral-flexion changed by 1.54°, from 31.54° to 33.08° from baseline to immediately after treatment and 0.74 ° from 33.08 to 33.82° from immediately after treatment to 24 hours follow-up. Left rotation changed by 1.39°, from 53.79° to 55.18° from baseline to immediately after treatment and −1.28° from 55.18° to 53.90° from immediately after treatment to 24 hours follow-up.	Low risk
							Right rotation changed by 1.64°, from 55.28° to 56.92° from baseline to immediately after treatment and 0.46° from 56.92° to 57.38° from immediately after treatment to 24 hours follow-up. In the HVLA group cervical flexion changed by 4.97°, from 55.08° to 60.05° from baseline to immediately after treatment and 0.31° from 60.05° to 60.36° immediately after treatment to 24 hours follow-up. Cervical extension changed by 7.8°, from 48.51 to 56.31 from baseline to immediately after treatment and −2.1° from 56.31° to 54.21° from immediately after treatment to 24 hours follow-up. Left lateral-flexion changed by 5.64°, from 32.77° to 38.41° from baseline to immediately after treatment and – 0.15 ° from 38.41 to 38.26° from immediately after treatment to 24 hours follow-up. Right lateral flexion changed by 4.72, from 33.33° to 38.05° from baseline to immediately after treatment and 1.23 ° from 38.05 to 36.82° from immediately after treatment to 24 hours follow-up. Left rotation changed by 9.29°, from 53.33° to 62.62° from baseline to immediately after treatment and −1.03° from 62.62° to 61.59° from immediately after treatment to 24 hours follow-up. Right rotation changed by 6.51°, from 55.54° to 62.05° from baseline to immediately after treatment and −1.18° from 62.05° to 60.87° from immediately after treatment to 24 hours follow-up. In the mobilization group, cervical flexion changed by 3.84°, from 59.44° to 63.28° from baseline to immediately after treatment and −1.64° from 63.28° to 61.64° immediately after treatment to 24 hours follow-up. Cervical extension changed by 2.51°, from 58.82 to 61.33 from baseline to immediately after treatment and −3.18° from 61.33° to 58.15° from immediately after treatment to 24 hours follow-up.	
							Left lateral-flexion changed by 3.49°, from 36.41° to 39.90° from baseline to immediately after treatment and −2.05° from 39.90 to 37.85° from immediately after treatment to 24 hours follow-up. Right lateral-flexion changed by 2.72, from 38.97° to 41.69° from baseline to immediately after treatment and 1.38 ° from 41.69° to 40.31° from immediately after treatment to 24 hours follow-up. Left rotation changed 4.15° from 57.54° to 61.69° from baseline to immediately after treatment and 2.16° from 61.69° to 63.85° from immediately after treatment to 24 hours follow-up. Right rotation changed by 6.26°, from 58.41° to 64.67° from baseline to immediately after treatment and 0.43° from 64.67° to 65.10° from immediately after treatment to 24 hours follow-up. Significant increase in CROM for the HVLA group from baseline Immediate: HVLA compared to control: significant increase in flexion, extension, left and right lateral flexion, and left rotation [p < 0.05]. HVLA compared to mobilization: significant increase in extension and left rotation [p < 0.05]. 24 hours-follow up: HVLA versus control: significant improvement in flexion, left lateral flexion, and left rotation [p < 0.05]. HVLA compared to mobilization: no significant differences. Effect sizes between groups: Flexion: Control group vs manipulation group: Immediate: −0.1 24 hrs: −0.2 Control group vs mobilisation group: Immediate: −0.5 24 hrs: −0.3	
							Manipulation group vs mobilisation group: Immediate: −0.4 24 hrs: −0.1 Extension: Control vs manipulation: Immediate: −0.4 24 hrs: 0.3 Control vs mobilisation: Immediate: −0.8 24 hrs: 0.0 Manipulation vs mobilisation: Immediate: −0.4 24 hrs: −0.3 Left lateral flexion: Control vs manipulation: Immediate: −0.422 24 hrs: −0.549 Control vs mobilisation: Immediate: −0.687 24 hrs: −0.545 Manipulation vs mobilisation: Immediate: −0.200 24 hrs: 0.066 Right lateral flexion: Control vs manipulation: Immediate: −0.6 24 hrs: −0.4 Control vs mobilisation: Immediate: −1.2 24 hrs: −1.2 Manipulation vs mobilisation: Immediate: −0.5 24 hrs: −0.5 Left rotation: Control vs manipulation: Immediate: −0.6 24 hrs: −0.6 Control vs mobilisation: Immediate: −0.5 24 hrs: −0.8 Manipulation vs mobilisation: Immediate: 0.1 24 hrs: −0.2	
							Right rotation: Control vs manipulation: Immediate: −0.4 24 hrs: −0.3 Control vs mobilisation: Immediate: −0.6 24 hrs: −0.7 Manipulation vs mobilisation: Immediate: −0.3 24 hrs: −0.5 No MDC was reported	
Young et al. [2019]	Patients with cervical radiculopathy with or without neck pain HVLA group [n = 22: mean age 48.8 ± 11.5] Sham group [n = 21: mean age 43.1 ± 10.8]	Thoracic	HVLA to upper and midthoracic spine [C7-T3 and T4-T9] 1 Session	Sham manipulation to upper and midthoracic spine [C7-T3 and T4-T9] [Same position, open hands on patient, no thrust] 1 session	Immediately posttreatment 48-72 hours posttreatment	Active cervical ROM [flexion, extension, rotation, and side-bending to symptomatic and asymptomatic side] Goniometer	Immediately after treatment, the HVLA group had greater cervical flexion [10.8°], extension [10.0°], and rotation to the symptomatic side [14.2°] and asymptomatic side [9.2°] compared to the sham group. 48–72 hours after treatment the HVLA group had greater cervical flexion [13.7°], extension [11.1°], rotation to symptomatic side [13.9°] and asymptomatic side [11.4°], side bending to symptomatic side [8,6°] Significant group by time interaction for flexion, extension, rotation to the symptomatic and asymptomatic side, side-bending to symptomatic side [all p < .05] with moderate to large effect sizes. Effect sizes between groups given by Young et al.: Flexion: Baseline to immediate: 0.7 Baseline to 48–72 hours: 0.8 Extension: Baseline to immediate: 0.8 Baseline to 48.72 hours: 1.0 Side bending symptomatic side Baseline to immediate: 0.3 Baseline to 48–72 hours: 0.9	Low risk
							Side bending asymptomatic side: Baseline to immediate: 0.2 Baseline to 48–72 hours: 0.2 Rotation symptomatic side Baseline to immediate: 1.3 Baseline to 48–72 hours: 1.4 Rotation asymptomatic side: Baseline to immediate: 0.7 Baseline to 48–72 hours: 0.8 MDC was mentioned from other studies	
González-Iglesias et al. [2009b]	Patients with acute mechanical neck pain <1 month HVLA group [n = 23: mean age 34 ± 4] Control group [n = 22: Mean age 35 ± 6]	Thoracic	HVLA to the thoracic spine [maximum 2 thrusts] 1x/week for 3 weeks AND Electrotherapy/thermal program [superficial thermotherapy, TENS, 20 minutes] 5 sessions within 3 weeks	Electrotherapy/thermal program [superficial thermotherapy, TENS, 20 minutes] 5 sessions within 3 weeks	Visit 5 [i.e., 3 days after the last HVLA, the same day as the last electro/thermal program] 2 weeks and 4 weeks posttreatment	Active cervical ROM [flexion, extension, right/left lateral flexion, right/left rotation] Goniometer	From baseline to final treatment: Cervical flexion changed by 0.4 ° in the electro/thermal program and 12.4° in the HVLA group. Cervical extension changed by 0.2 ° in the electro/thermal program and 11.6° in the HVLA group. Cervical right rotation changed by −1.4 ° in the electro/thermal program and 11.1° in the HVLA group. Cervical left rotation changed by −0.4 ° in the electro/thermal program and 10.8 ° in the HVLA group. Cervical right lateral flexion changed by 0.4 ° in the electro/thermal program and 9.7 ° in the HVLA group. Cervical left lateral flexion changed by- 0.8 ° in the electro/thermal program and 10.0 ° in the HVLA group. From baseline to 2-week follow-up: Cervical flexion changed by 3.1 ° in the electro/thermal program and 11.2 ° in the HVLA group. Cervical extension changed by −0.6 ° in the electro/thermal program and 6.4 ° in the HVLA group. Cervical right rotation changed by −4.3 ° in the electro/thermal program and 7.8 ° in the HVLA group. Cervical left rotation changed by −2.1° in the electro/thermal program and 6.4 ° in the HVLA group. Cervical right lateral flexion changed by 0.8 ° in the electro/thermal program and 9.5 ° in the HVLA group. Cervical left lateral flexion changed by 1.7 ° in the electro/thermal program and 7.6 ° in the HVLA group.	Some concern
							Improvement in all cervical ROM in thrust vs non-thrust group Overall group x time interaction was statistically significant [p < 0.05]. No post-hoc tests were reported that would allow assessing whether changes at 2 and 4 weeks were still significant but values that are almost as good as directly after intervention would suggest so. No MCD reported Effect sizes between groups: Flexion: 2.7 Extension: 1.9 Right rotation: 1.8 Left rotation: 2.4 Right lateral flexion: 1.9 Left lateral flexion: 2.4	
Puntumetakul et al. [2015]	Patients with chronic mechanical neck pain > 3 months Single HVLA [n = 16: mean age 25.9 ± 9.7] Multiple HVLA [n = 16] mean age 26.6 ± 8.2] Control group [n = 16: mean age 27.1 ± 7.5]	Thoracic	Single HVLA to thoracic spine [T6-T7] OR Multiple HVLA to thoracic spine [T6-T7 and next segmental restrictions] 1 session	Prone lying 2 minutes [with therapist‘s hands over the level of the thoracic zygapophyseal joint] 1 session	24 hours posttreatment 1 week posttreatment	Active cervical ROM [flexion, extension, right/left lateral flexion, right/left rotation] CROM device	No group-specific range of motion was reported. Meaningful comparison of single HVLA vs. control, multiple HVLA vs. control. No significant within-group difference for single or multiple HVLA at 24 hours and 1 week follow-up compared to baseline. 24 hours follow up: Multiple HVLA vs. control: ROM in right rotation significantly increased [p < 0.05] 1 week follow-up: Single HVLA versus control: ROM in left lateral flexion and flexion significantly increased [p < 0.05]. Effect sizes could not be calculated due to unpublished detailed follow-up data. No MCD reported	Some concern
Hanney et al. [2017]	Healthy participants HVLA group [n = 34: mean age 23.6 ± 2.8] Stretching group [n = 34: mean age 23.8 ± 3.6] Control group [n = 34: Mean age 23.4 ± 3.0]	Thoracic	HVLA to cervicothoracic spine [T1, both sides, maximum of 2 thrusts each side] 1 session	Stretching group: manual upper trapezius stretches [2x30 seconds on each side] OR Control group: seated position for 3–5 minutes 1 session	Immediately posttreatment [within 5 minutes]	Active cervical ROM [flexion, extension, right/left lateral flexion, right/left rotation] CROM device	Pre-intervention/ post- intervention HVLA group: Cervical flexion changed by 1.47°, from 56.82° to 58.29°, cervical extension changed by −1.94°, from 72.29 ° to 70.35 °. Left cervical lateral flexion changed by 1.94 °, from 47.12° to 49.06°. Right cervical lateral flexion by 0.65°, from 49.82° to 50.47°. Left cervical rotation changed by 0.76°, from 70.59° to 71.35°. Right cervical rotation changed by – 0.91°, from 71.65° to 72.65°. Pre-intervention/ post-intervention control group: Cervical flexion changed by −2.24°, from 56.65° to 54.41°. Cervical extension changed by −5.7°, from 69.94 ° to 64.24 °. Left cervical lateral flexion changed by −0.88°, from 48.06° to 47.18°. Right cervical lateral flexion by −3.11°, from 51.82° to 48.71°. Left cervical rotation changed by −2.24°, from 70.59° to 78.35°. Right cervical rotation changed by – 0.88°, from 69.00° to 68.12°. Significant time X group effect for extension and cervical lateral flexion left and right. Effect sizes between groups Flexion: −0.4 Extension: −0.5 Left lateral flexion: −0.2 Right lateral flexion: −0.1 Left rotation: 0.3 Right rotation: 0.4 MDC was mentioned, values from other studies considered.	Some concern
Dunning et al. [2012]	Patients with neck pain HVLA group [n = 56: mean age 41.5 ± 11.9] Mobilization group [n = 51: mean age 42.7 ± 13.9]	Cervical and thoracic	HVLA to upper cervical spine [C1/2, both sides] and upper thoracic spine [T1/2] AND advice 1 session	Mobilization to upper the cervical spine [C1/2, grade IV, 30 seconds on each side] and upper thoracic spine [T1/2] AND advice 1 session	48 hours posttreatment	Passive ROM of C1/2 rotation [flexion rotation test = FRT] CROM device	Within-group change score [baseline to 48 hours follow-up] for HVLA group 8.4° for right FRT, and 5.9° for left FRT. Within-group change score [baseline to 48 hours follow-up] for mobilization group 3.5° for right FRT and 2.4 ° for left FRT. Between-group differences were statistically significant for right FRT [p < 0.001] and left FRT [p < 0.004]. No MDC was reported for ROM. Effect sizes between groups: FRT right: 0.4 FRT left: 0.7	Low risk

**Table 3:** Effects of HVLA thrust on facet joint gapping and spinal stiffness.

Autor, Year	Participants [A]Symptomatic Age Number	Treatment region/ Region of interest	Intervention	Control	Time to assessment [relative to intervention]	Biomechanical outcome parameters	Main biomechanical Results	RoB Rating
Cramer et al. [2002]	Students without a history of significant lower back pain 2 HVLA groups: Group 2 [n = 16] Group 3 [n = 16] 2 control groups: Group 1 [n = 16] Group 4 [n = 16] Age: 22–30 yrs Average age: 24.9 ± 1.8	Lumbar	HVLA to the lumbar spine [side-posture on left, L3-S1, multiple thrusts] Group 2: Neutral MRI, HVLA, neutral MRI Group 3: Neutral MRI, HVLA, side-posture MRI 1 session	Side-posture positioning [SPP] Group 1: Neutral MRI, SPP, side-posture MRI Group 4: Neutral MRI, very brief SPP, neutral MRI 1 session	Posttreatment	Gapping difference of L3/L4, L4/L5 and L5/S1 Z-joints in MRI	The difference between group 1 and group 4 is 1.18 mm [p < 0.0001], with the side-posture position group showing a larger gap. The difference between group 3 and group 4 is 1.89 mm [p < 0.0001], with group 3 showing a larger gap. A similar pattern was observed between group 3 and group 1, with a mean difference of 0.71 mm [p = 0.047]. There was no significant difference between group 2 and group 4 [mean difference 0.12 mm, p = 1.00]. No effect sizes could be calculated due to lack of detailed reporting of ROM data. MDC was reported [based on a preliminary study but only in relation to sample size calculation]: 0.5 mm	Low risk
Cramer et al. [2012]	Healthy subjects without a history of low back pain HVLA group [n = 30] Control group [n = 10] Age: 18–30 yrs	Lumbar	HVLA to lumbar spine [side-posture on left, L3-S1, 2 thrusts] initial MRI in neutral, second MRI in side-posture 1 session	Side-posture positioning initial MRI in neutral, second MRI in side-posture 1 session	Immediately post-treatment	Gapping difference of L3/L4, L4/L5 and L5/S1 Z-joints in MRI	The mean gapping difference for all upside joints was statistically significantly larger than that of downside joints [0.7 mm vs. – 0.2 mm]. HVLA upside joints gapped more than SPP upside joints [0.7 vs 0.5 mm, p = 0.03]. HVLA downside joints space decreased less than SPP downside joints [−0.1 vs −0.4 mm, p = 0.01]. No effect sizes could be calculated due to lack of detailed reporting of ROM data. No MDC was reported.	Some concern
Cramer et al. [2013]	Patients with acute low back pain< 6 weeks 2 HVLA groups: Group 2 [n = 28: mean age 42.7 ± 10.3] Group 3 [n = 28: mean age 43.7 ± 12.7] 2 control groups: Group 1 [n = 28: mean age 44.2 ± 12.7] Group 4 [n = 28: mean age 47.6 ± 10.0]	Lumbar	HVLA to the lumbar spine [side-posture, most painful side up, L3-S1, multiple thrusts] Group 2: Neutral MRI, HVLA, neutral MRI Group 3: Neutral MRI, HVLA, side-posture MRI	Side-posture positioning [SPP] Group 1: Neutral MRI, SPP, side-posture MRI Group 4: Neutral MRI, SPP, neutral MRI	MRI 1: scan before any treatment began and right after the treatment MRI 2: scan before treatment and right after In between both MRI sessions were two weeks with treatment at a chiropractor	Greatest gapping difference of L4/L5 or L5/S1 Z-joint in MRI before and after SMT or SPP	First Appointment: Group 1 had a greater gapping pre-post than the three other groups [Mean differences: Group 1 = 1.09 mm, Group 2 = 0.24 mm, Group 3 = 0.19 mm, Group 4 = 0.71 mm, p = 0.001]. Second Appointment: Group 3 had greater gapping pre-post than the other three groups [Mean differences: Group 1 = 0.65 mm, Group 2 = 0.18 mm, Group 3 = 0.76 mm, Group 4 = 0.44 mm, p = 0.005] Use of a crossover design for the MRI 2 protocol. No effect sizes could be calculated due to lack of detailed reporting of ROM data. No MDC was reported.	Low risk
Pagé and Descarreaux [2019]	Chronic thoracic pain ≥ 3 months in T6-8 region 3 HVLA groups: Group 1 [n = 20: mean age 41.5 ± 13.8] Group 2 [n = 22: mean age 37.5 ± 13.5] Group 3 [n = 21: 37.2 ± 11.1] Control group [n = 18: 35.8 ± 13.7]	Thoracic spine	HVLA to thoracic spine [single thrust at T7, applied with apparatus] Group 1 135N peak force 125ms impulse duration 920N/s rate of application Group 2 250N peak force 250ms impulse duration 1840N/s rate of application Group 3 250N peak force 250ms impulse duration 920N/s rate of application 3 sessions over 2–3 weeks	Control group: Rested quietly for 5 minutes 3 sessions over 2–3 weeks	Posttreatment at each treatment session 6-8 days after last treatment	Spinal stiffness	The global stiffness showed a mean decrease of −0.01 N/mm at T6, −0.25 N/mm at T7, and −0.32 N/mm at T8. Changes were −0.04 N/mm, −0.25 N/mm, and −0.33 N/mm for terminal stiffness. No statistically significant difference in any of the secondary outcomes between the four groups [all p values < 0.005]. Spinal stiffness at T7 and T8 was reduced in all groups [including the control group]. No effect sizes could be calculated due to lack of detailed reporting of stiffness data. Strength: Between-group comparison using a randomized sequence	Some concern

### Local effects of HVLA thrusts on ROM

#### Effects of cervical HVLA thrusts on cervical ROM.

16 (47%) studies investigated the effects of cervical HVLA thrust on cervical ROM in different patient populations, e.g., mechanical neck pain, chronic neck pain, and tension-type headache [32–36,39,41,55–60,64–66]. Seven studies (43.7%) were rated low risk and nine (56.2%) as some concerns [55–60,64–66]. Nine (56.2%) of these studies showed an increase in cervical range of motion (4 low-risk and 5 some concerns) [32,33,39,41,55,56,58,60,64]. Included studies were heterogeneous in terms of the movement planes considered, number of movement planes where an improvement was observed, treatment frequency, and time of measurement after HVLA thrust: treatment approaches ranged from one session with immediate post-treatment measurement to a maximum of eight sessions over four weeks [60] (Table 1).

#### Effects of thoracic HVLA thrusts on thoracic ROM.

Two studies (5.8%) with low risk of bias investigated the effect immediately after the thoracic spine manipulation on thoracic ROM. One [2.9%] study showed an immediate post-treatment improvement on active thoracic flexion but not on extension, of a single T9 HVLA thrust compared to a sham intervention in a sample of 22 participants. The other study (2.9%) [N = 52] investigated active thoracic excursion (i.e., full flexion to full extension) in patients with subacromial impingement syndrome of the shoulder and found no difference between a total of 6 HVLA thrusts applied to the upper, middle, and lower thoracic spine in a single session compared to a sham intervention [38] (Table 1).

#### Effects of lumbar HVLA thrusts on lumbar ROM.

Two studies (5.8%) rated some concerns of bias, investigated lumbar spine HVLA thrusts effects on lumbar ROM [63,67]. One study (2.9%) included 330 participants with chronic non-specific LBP at level L4/5, in three treatment arms, and measured the effects on active ROM using the modified Schober test. The patients underwent twelve treatment sessions (3x/week for four weeks) including lumbar spine HVLA thrusts plus laser plus exercises, or laser and exercises without HVLA thrust, or exercises only. The modified Schober test showed an improvement for the HVLA thrust group compared to the two other groups [63]. Another study (2.9%) with 14 participants with lumbar spinal stenosis investigated the effects of bilateral lumbar HVLA thrusts on active ROM [flexion, extension, left lateral flexion, right lateral flexion, left rotation and right rotation]. No within or between group differences pre- and post-treatment were reported [67] (Table 1).

### Regional effects of HVLA thrusts on ROM

#### Effects of thoracic HVLA thrusts on cervical ROM.

Eight studies investigating the effects of thoracic spine HVLA thrusts on cervical ROM showed an increase in cervical ROM [28,30,37,40,61,62,68,69]. Four (50%) were rated as low risk of bias [28,30,37,40] and four (50%) as having some concerns for risk of bias [61,62,68,69]. Five (62.5%) included acute or chronic neck pain patients [28,30,37,68,69], two (25%) included cervical radiculopathy patients with or without neck pain [40,61], and one (12.5%) included healthy participants [62]. One (12.5%) study only measured cervical rotation and consequently only reported an increase of rotation [28]. The other studies (87.5%) measured all planes of cervical ROM, with two (25%) reporting increased ROM in all planes [30,68] and five (62.5%) in different cervical planes [37,40,61,62,69]. Six studies (75%) measured immediately post-treatment [28,37,40,61,62,68], and two studies (25%) reported an increase of cervical ROM lasting up to one [30,69] or two weeks [68]. Treatment approaches ranged from one session with one to two HVLA thrusts on one site [28,37,61,62,69] or multiple sites [40,69] to multiple sessions of HVLA thrusts [68] (Table 2).

#### Effects of thoracic and cervical HVLA thrusts on cervical ROM.

One study with low risk of bias compared HVLA thrusts with mobilization of the upper cervical and the upper thoracic region on the passive range of cervical rotation of C1/2 in a neck pain population and reported a significantly greater increase in both rotation directions for the HVLA thrust compared to the mobilization group 48 hours after the intervention [31] (Table 2).

### Effects of HVLA thrusts on facet joint gapping

Three studies compared the effects of lumbar side-posture HVLA thrusts with pure side-posture positioning on gapping of the lumbar facet joints as measured by MRI before and after application of the HVLA thrusts [26,27,53]. Two studies (66%) were rated as low risk of bias and one (33%) as having some concerns for risk of bias. Two larger follow-up studies, also in healthy participants, showed statistically significant differences in gapping between the subjects receiving side-posture HVLA thrusts and those receiving side-posture positioning only, but only when the MRI following HVLA thrusts was conducted in side-posture. In patients with acute LBP (N = 112) [26], at onset, pure side-positioning resulted in more gapping than HVLA thrusts [delivered with the most painful side positioned up] followed by side-positioning. In contrast, after two weeks of chiropractic treatment, facet joint gapping after HVLA thrusts was greater than after side-positioning (Table 3).

### Effects of HVLA thrust on spinal stiffness

One study rated some concerns for risk of bias and investigated spinal stiffness after the application of IAM HVLA thrusts to T7 in chronic thoracic pain patients [54]. This four-arm trial (N = 81) included three HVLA thrust groups using different dosages of applied force magnitude, impulse duration, and rate of force application, and a control group. The treatment regime was three sessions over two to three weeks. Spinal stiffness was measured before and after HVLA thrusts over the spinous processes of T6, T7, and T8. Displacement of vertebrae was measured using an indenter device during exhalation using the following procedure: after application of a posterior-to-anterior load of 5N on the spinous process, the load was gradually increased with an 18 N/s rate of force application to 45 N. Terminal and global spinal stiffness coefficients were calculated using the force and displacement data recorded during each spinal stiffness trial. Terminal stiffness was defined as the ratio of the load divided by the displacement between 10 and 45 N, and global stiffness was defined as the slope of the straight-line best fitting the data over the same load interval. Stiffness was reduced after the intervention in all groups, including the control group, but no statistically significant difference was found between the groups (Table 3).

### Relationship between biomechanical and patient-rated outcomes

Sixteen (47%) of the included studies used patient-rated outcome measures such as pain [visual analogue pain scale, numeric pain rating scale, 9-point faces pain scale] [28,30,34,37,41,57,59,60,64–66,68,69] and/or disability [neck pain and disability scale, neck disability index, Northwick Park neck pain questionnaire, jaw functioning limitation scale] [30,32,40,41,56,64,66,69] as primary outcome measures. One study, rated some concerns for risk of bias, statistically linked these patient-rated outcomes to biomechanical measures. This study reported an association between neck pain at rest and the improvement in ROM, i.e., the greater the increase in ROM, the greater the improvement in neck pain.

## Discussion

This systematic review found evidence in support for local effects of HVLA thrusts to the cervical spine on cervical ROM and for regional effects of thoracic HVLA thrusts on cervical ROM. This indicates that cervical ROM can be increased by HVLA thrusts to either the cervical or the thoracic spine. In contrast, evidence regarding local effects of HVLA thrusts to the thoracic and lumbar spine remains inconclusive. Similarly, it is not yet clear whether HVLA thrusts lead to facet joint gapping or reduce spinal stiffness. Most studies investigated pain and/or disability as patient-reported outcomes, but only one analyzed their relationship to the biomechanical measures.

### Local effects of HVLA thrusts on ROM

The finding of HVLA thrusts increasing cervical spinal ROM is in line with previous systematic reviews [19,20]. Although the heterogeneity of the studies regarding treatment approaches and participant characteristics hinders comparison, there appears to be an immediate effect of HVLA thrust to the cervical spine on increased cervical ROM. This also holds true when only considering studies delivering HVLA thrust-SMT to the upper cervical spine: the study that did not show an effect included patients with temporomandibular joint dysfunction [66], while the positive studies included patients with tension-type headache [32], cervicogenic headache [36] or chronic neck pain [58]. Patients with temporomandibular joint dysfunction might not necessarily have upper cervical joint dysfunctions and adding HVLA-SMT to the comparison treatment of suboccipital release and exercise might not change the biomechanical results. Overall, as studies differed with relation to measuring active or passive ROM and with relation to which planes were measured, no clear pattern emerged of which movement planes of cervical ROM are affected most or most often by HVLA thrust-SMT directed to the cervical spine.

As for the two studies [29,38] on the immediate, local effects of a single thoracic HVLA thrusts applied to the thoracic spine compared to a sham intervention. Ditcharles and colleagues applied a single thrust to T9 in patients with subacromial pain and showed an increase of thoracic flexion as measured by inclinometer [assessor-blinded] [29]. Kardouni and colleagues applied a total of six thoracic thrusts to young healthy adults and did not report any effect on thoracic spine extension and thoracic excursion using an electromagnetic motion capture system [38]. Thus, given the fact that only electromagnetic studies were available for the local effects of HVLA thrusts on the thoracic spine, the local effect of thoracic spine HVLA on thoracic ROM remains unclear and underlines the notion that research on the thoracic spine is scarce [70]. Two studies examining the local effects of HVLA thrusts on the lumbar spine showed divergent findings [63,67]. The larger study’s HVLA group demonstrated a significant reduction in pain and an improvement in function [63]. The smaller study did not find any effect of a single HVLA thrusts in patients with spinal canal stenosis [67]. In addition to being underpowered, as suggested by the authors, it is conceivable that it is more difficult in patients with spinal canal stenosis to increase ROM. Thus, several factors might be contributing to the divergent findings, including variations in the number of interventions administered (three session over four weeks vs. one single session with one single thrust), as well as differences in the sample size (n = 330 vs. n = 14) and type of the study populations (patients with uncomplicated chronic low back pain, lumbago, sciatica, with pain localized in L4/L5 vs. degenerative lumbar stenosis).

### Regional effects of HVLA thrusts on ROM

Eight studies investigating the effects of thoracic spine HVLA thrusts on cervical range of motion reported an improvement of cervical ROM, which is in line with findings of a previous systematic review [21]. A possible explanation for this finding might be that functional disturbances in the upper thoracic spine can lead to decreased muscle strength [71] and impaired ROM in the cervical spine. Thus, treating such functional disturbances in the upper thoracic spine by manual therapy to restore normal biomechanical function might normalize cervical ROM [71–74]. In addition, other phenomena might be contributing to the improvement of cervical ROM in response to thoracic HVLA thrusts, including those directed at the mid-thoracic spine. In particular, DNIC (diffuse noxious inhibitory controls)-like phenomena or non-specific treatment effects might be involved.

### Effects on facet joint gapping and spinal stiffness

Inactivity, asymmetrical loads, and injury can lead to aberrant facet joint motion, hypomobility, and adhesion formation [14,23]. Thus, gapping the facet joints and breaking up adhesions might be one of the mechanisms of action of HVLA thrusts [14]. The three studies, all conducted by the same research group, investigated the effects of lumbar HVLA thrusts on gapping of the facet joints in an asymptomatic [27,53] and symptomatic LBP study population [26] provided some evidence that HVLA thrusts immediately gaps the lumbar facet joints, but only if followed by side-posture positioning, which suggests a short-lived effect.

A decrease in spinal stiffness has been shown in small studies after HVLA thrusts and has been related to changes in ROM, pain, pressure pain threshold, and spinal tissue behavior, e.g., by relaxation of the spinal connective tissues and/or changed motor reflexes [75]. However, the single study on spinal stiffness included in this review did not confirm these findings [54]. It has to be considered that posterior to anterior stiffness measurements are influenced by multiple factors — including soft tissue compliance and joint morphology — and do not provide a direct quantification of intrinsic segmental stiffness. [76–78].

### Clinical considerations and recommendations for future studies

Interestingly, only one [58] of the included studies on ROM of any spinal region reported whether ROM was altered in the respective study population compared to norm values at baseline. This is a major shortcoming and hampers the interpretation of studies investigating the effect of a manual treatment that aims at improving ROM. Nevertheless, four articles refer to the minimal detectable change [MDC]: three studies report changes in ROM higher than the MDC [28,58,64] and one study changes lower than the MDC [40]. Future studies would benefit from quantifying a possible restriction of ROM beforehand and testing whether HVLA thrusts normalized ROM, considering the MDC or an alternative method to account for repeated measures effects. Furthermore, only one study investigated a possible relationship between biomechanical and patient-related outcomes such as pain or disability. For future studies, the incorporation of those links is strongly recommended and as long as such a link is not established, an increase in ROM alone should only be interpreted as a proxy for a successful outcome, when (painless) restricted ROM is one of the patients’ main complaints.

### Limitations

In general, the RoB tool presents challenges because it focuses only on specific domains and does not assess the overall quality of the study [79]. The RoB tool, used as a checklist, aids in quality rating through its built-in algorithm while also allowing assessors to adjust the rating. However, it is a complex tool [80], that should only be used after intensive training [80], as was done in the present study. Nevertheless, the group of raters was heterogeneous in terms of expertise in conducting systematic reviews, and not all raters were involved in all review stages during the updates. Additionally, the literature search was limited to the English language which may have excluded some relevant studies. Lastly, due to the high heterogeneity of data with regard to participant characteristics, intervention frequency, and outcomes, no meta-analysis could be performed.

## Conclusions

The main finding of this review, based on equal numbers of studies rated low-risk and some concerns, is that HVLA thrusts, applied to the cervical or the thoracic spine, increases cervical ROM, which is likely of clinical significance. However, the results on the local effects of an HVLA thrusts to the thoracic and lumbar spine, as well as on the effects of an HVLA thrusts on facet joint gapping and spinal stiffness, remain inconclusive. Future studies should quantify the outcome measures at baseline and assess potential relationships between the biomechanical effects of an HVLA thrust and clinical outcomes.

## Supporting information

S1 File Characteristics of excluded studies. (DOCX)

S2 File Search strategy. (PDF)

S3 File PRISMA checklist. (DOCX)
