# Supplementary material for: Systematic review on biomechanical effects of high-velocity, low amplitude spinal manipulation
Source: PLoS One. 2025 Jul 18;20(7):e0328048. doi: 10.1371/journal.pone.0328048 (PMC12273944; doi:10.1371/journal.pone.0328048)
Supplement: S1 File — (DOCX) [file pone.0328048.s001.docx]

***Range of Motion C-spine treated, C-spine measured***

| **Article** | **Subject health status** | **Subject characteristics** | **Treatment region** | **Were OM assessors aware of intervention received by study participants (4.3)**  **Yes/no**  **PY (perhaps yes)**  **PN (perhaps no)** | **Outcome**  **Active range of motion / passive range of motion** | **Within group comparison**  **Pre-post** | **Between group comparison**  **Pre-pre**  **Post-post** | **Mixed design**  **Pre-post between groups: yes/no** | **Positive result reported: yes/no**  **Time point after intervention: specify;**  **N/A**  **Effect size (ES) reported?**  **Yes, no** | **Negative result reported**  **Time point after intervention**  **N/A** | **RoB Rating** |
| --- | --- | --- | --- | --- | --- | --- | --- | --- | --- | --- | --- |
| Pikula, J. R. (1999). | symptomatic | Patients with acute unilateral neck pain and stiffness < 2 weeks; | Cervical | Yes | Active range of motion | Pre-post |  | ANOVA  Post hoc Tukey test | Yes  Immediate  ES: no |  | High risk |
| Saayman, L., et al. (2011) | symptomatic | Patients with cervical facet joint dysfunction between 1 and 12 months | Cervical | Yes | Active range of motion  2 diff measures | Pre-post | Pre-pre  Post—post?  Kruskal Wallis and Mann Whitney) | Multiple regression models with Bonferroni adjustment | Yes  1 week after last treatment  Es: no |  | High risk |
| Lin J. et al, (2013) | symptomatic | Mechanical neck pain | Cervical | no | Active range of motion | Pre-Post one-way repeated measures ANOVA | Pre-pre independent t-test | Yes Linear mixed models | Yes 4.4  LM group better  Yes: immediately and 3 months follow-up  Yes: For NPQ | Yes  Immediate post treatment and 3-month follow-up (Table 2) Results LMM | High risk |

***Range of Motion T-spine treated, T-spine measured***

| **Article** | **Subject health status** | **Subject characteristics** | **Treatment region** | **Were OM assessors aware of intervention received by study participants (4.3)**  **Yes/no**  **PY (perhaps yes)**  **PN (perhaps no)** | **Outcome**  **Active range of motion /**  **passive range of motion** | **Within group comparison**  **Pre-post** | **Between group comparison**  **Pre-pre**  **Post-post** | **Mixed design**  **Pre-post between groups: yes/no** | **Positive result reported: yes/no**  **Time point after intervention: specify;**  **N/A**  **Effect size reported?**  **Yes, no** | **Negative result reported**  **Time point after intervention**  **N/A** | **RoB Rating** |
| --- | --- | --- | --- | --- | --- | --- | --- | --- | --- | --- | --- |
| Gavin, D. (1999) | asymptomatic | Asymptomatic volunteers; | Thoracic | No | Active range of motion | Yes | No | No: ANOVAs performed for each of the three groups separately | Yes – pre-post but unclear for which group (control group per table, SMT group per text)  Probably Immediately after intervention  ES: no |  | High risk |
| Griffiths, F (2019) | asymptomatic | Asymptomatic osteopathic students | Thoracolumbar junction | No | Active range of motion | Yes, pre-post | No | One-way ANOVA using change data from post subtracted by pre-ROM data | Yes.  Yes, post intervention  ES: Yes | Yes: Control versus sham no difference. | High risk |

***Range of Motion T-spine treated, C-spine measured***

| **Article** | **Subject health status** | **Subject characteristics** | **Treatment region** | **Were OM assessors aware of intervention received by study participants (4.3)**  **Yes/no**  **PY (perhaps yes)**  **PN (perhaps no)** | **Outcome**  **Active range of motion /**  **passive range of motion** | **Within group comparison**  **Pre-post** | **Between group comparison**  **Pre-pre**  **Post-post** | **Mixed design**  **Pre-post between groups: yes/no** | **Positive result reported: yes/no**  **Time point after intervention: specify;**  **N/A**  **Effect size reported?**  **Yes, no** | **Negative result reported**  **Time point after intervention**  **N/A** | **Risk of Bias (RoB) Rating** |
| --- | --- | --- | --- | --- | --- | --- | --- | --- | --- | --- | --- |
| Lau, H. M. C., et al. (2011). | symptomatic | Patients with chronic mechanical neck pain > 3 months | Thoracic | No | Active range of motion | Pre-post | Pre-pre | ANOVA with differences pre-post | Yes  Immediately, after 3 and 6 months  ES: mentioned in methods section, but no results found? |  | High risk |

***Range of Motion various approaches***

| **Article** | **Subject health status** | **Subject characteristics** | **Treatment region** | **Were OM assessors aware of intervention received by study participants (4.3)**  **Yes/no**  **PY (perhaps yes)**  **PN (perhaps no)** | **Outcome**  **Active range of motionis /**  **passive range of motion** | **Within group comparison**  **Pre-post** | **Between group comparison**  **Pre-pre**  **Post-post** | **Mixed design**  **Pre-post between groups: yes/no** | **Positive result reported: yes/no**  **Time point after intervention: specify;**  **N/A**  **Effect size reported?**  **Yes, no** | **Negative result reported**  **Time point after intervention**  **N/A** | **RoB Rating** |
| --- | --- | --- | --- | --- | --- | --- | --- | --- | --- | --- | --- |
| **Yoshida, 2024** | **symptomatic** | **Patients with mechanical neck pain** | **Thoracic** | **Yes** | **Active cervical and thoracic range of motion** |  |  | **Linear mixed model with group and measurement time points as factors.** | **Yes**  **Thoracis rotation: immediately, one week and four weeks after intervention** | **Yes**  **Neck rotation: one and four weeks** | **High risk** |
| Martel, J., et al. (2011). | symptomatic | Patients with non-specific chronic neck pain > 12 weeks; | Cervical and thoracic | Yes | Active range of motion cervical spine | Pre-post (dependent t-test) | Pre-pre (one way ANOVA) | ANCOVA, with treatment and time intervals representing the main factors | No  Every 2 months within 10 months  ES: No | Yes  ANCOVA (adjusted for gender and pain improvement in the symptomatic phase) did not yield significant between group differences | High risk |
| Vavrek, D., et al. (2010). | symptomatic | Patients with at least 5 cervicogenic headaches per month for > 3 months; | Cervical and/or thoracic | no | Active range of motion cervical spine | Pre-post | Pre-pre | Linear regression | no | Yes  End of treatment  ES: no | High risk |

**Stiffness/Animal**

| **Article** | **Subject health status** | **Subject characteristics** | **Treatment region** | **Within comparison** | **Between comparison** | **Mixed design** | **Positive effect**  **Short term**  **Long term** | **No positive effect**  **Short term**  **Long term** | **RoB Rating** |
| --- | --- | --- | --- | --- | --- | --- | --- | --- | --- |
| Haussler, K. K., et al. (2010). | asymptomatic | Actively ridden horses without current history of acute back problems; | Thoracic and lumbar | Pre-post | Pre- pre  Post-post  (bin mir da nicht sicher) | Mixed effect linear regression model with horse as random effect | Trend for interaction group*time (p=0.06)  After 3 weeks (final treatment)  ES: No |  | High risk |

**Stiffness / Human**

| **Article** | **Subject health status** | **Subject characteristics** | **Treatment region** | **Within comparison** | **Between comparison** | **Mixed design** | **Positive effect**  **Short term**  **Long term** | **No positive effect**  **Short term**  **Long term** | **RoB Rating** |
| --- | --- | --- | --- | --- | --- | --- | --- | --- | --- |
| Nim C., et al., (2020) | symptomatic | Persistent LBP (> 3 months) | T12-L1 | Pre-post (table 3) | Pre-post (table 3) | Linear mixed model | None | No significance within or between groups for stiffness | High |
