# Supplementary material for: Systematic review on biomechanical effects of high-velocity, low amplitude spinal manipulation
Source: PLoS One. 2025 Jul 18;20(7):e0328048. doi: 10.1371/journal.pone.0328048 (PMC12273944; doi:10.1371/journal.pone.0328048)
Supplement: S2 File — (PDF) [file pone.0328048.s002.pdf]

Wednesday, March 22, 2023 3:18:08 PM

#	Query	Limiters/Expanders	Last Run Via	Results
S6	(S1 AND S2 AND S3) AND EM 20201020-	Limiters - Language: English, German<br>Expanders - Apply equivalent subjects<br>Search modes - Find all my search terms	Interface - EBSCOhost Research Databases<br>Search Screen - Advanced Search<br>Database - MEDLINE	127
S5	S1 AND S2 AND S3	Limiters - Language: English, German<br>Expanders - Apply equivalent subjects<br>Search modes - Find all my search terms	Interface - EBSCOhost Research Databases<br>Search Screen - Advanced Search<br>Database - MEDLINE	1,343
S4	S1 AND S2 AND S3	Expanders - Apply equivalent subjects<br>Search modes - Find all my search terms	Interface - EBSCOhost Research Databases<br>Search Screen - Advanced Search<br>Database - MEDLINE	1,394
S3	(MH "Pressure+") OR (MH "Zygapophyseal Joint") OR (MH "Biomechanical Phenomena+") OR (MH "Elastic Modulus") OR (MH "Elasticity+") OR (MH "Range of Motion, Articular+") OR (MH "Intervertebral Disc") OR (MH "Joint Capsule+") OR (MH "Torque") OR (MH "Torsion, Mechanical+") OR (MH "Stress, Mechanical") OR TI ("Range of Motion" OR ROM OR "Facet joint*" OR "Spinal tissue" OR Stiffness OR Displacement OR Crepitus OR Pressure OR Zygapophyseal OR cavitation OR "Spinal curvature*" OR "postur* chang*") OR TI (joint N3 (space OR motion OR movement OR gapping OR capsule)) OR TI (("soft	Expanders - Apply equivalent subjects<br>Search modes - Find all my search terms	Interface - EBSCOhost Research Databases<br>Search Screen - Advanced Search<br>Database - MEDLINE	1,563,848

	tissue" OR muscle OR vascular OR vessel OR arter* OR vein OR neural OR nerve) N6 (elongation* OR deformation* OR stretch* OR compression* OR tension* OR lengthening* OR distension* OR strain* OR prolongation* OR extension*)) OR AB ("Range of Motion" OR ROM OR "Facet joint*" OR "Spinal tissue" OR Stiffness OR Displacement OR Crepitus OR Pressure OR Zygapophyseal OR cavitation OR "Spinal curvature*" OR "postur* chang*") OR AB (joint N3 (space OR motion OR movement OR gapping OR capsule)) OR AB (("soft tissue" OR muscle OR vascular OR vessel OR arter* OR vein OR neural OR nerve) N6 (elongation* OR deformation* OR stretch* OR compression* OR tension* OR lengthening* OR distension* OR strain* OR prolongation* OR extension*))			
S2	(MH "Back+") OR (MH "Neck") OR TI (neck OR back OR cervical OR thoracic OR lumbar OR sacrum OR lumbosacral OR spine) OR AB (neck OR back OR cervical OR thoracic OR lumbar OR sacrum OR lumbosacral OR spine)	Expanders - Apply equivalent subjects<br>Search modes - Find all my search terms	Interface - EBSCOhost Research Databases<br>Search Screen - Advanced Search<br>Database - MEDLINE	1,004,239
S1	(MH "Musculoskeletal Manipulations") OR (MH "Manipulation, Chiropractic") OR (MH "Manipulation, Osteopathic") OR (MH "Manipulation, Orthopedic")	Expanders - Apply equivalent subjects<br>Search modes - Find all my search terms	Interface - EBSCOhost Research Databases<br>Search Screen - Advanced Search<br>Database - MEDLINE	14,197

OR (MH "Manipulation, Spinal") OR TI (spin* N3 manipulat*) OR TI ("high velocity" N3 "low amplitude") OR TI ("SMT" OR "HVLA") OR AB (spin* N3 manipulat*) OR AB ("high velocity" N3 "low amplitude") OR AB ("SMT" OR "HVLA")
